# Anthropogenic mortality of large mammals and trends of conflict over two decades in Nepal

**DOI:** 10.1002/ece3.9381

**Published:** 2022-10-03

**Authors:** Kedar Baral, Shivish Bhandari, Binaya Adhikari, Ripu M. Kunwar, Hari P. Sharma, Achyut Aryal, Weihong Ji

**Affiliations:** ^1^ School of Natural and Computational Science Massey University Auckland New Zealand; ^2^ Division Forest Office Pokhara Nepal; ^3^ Natural Science Society Kathmandu Nepal; ^4^ Tribhuvan University Institute of Forestry Kaski Nepal; ^5^ Pokhara Zoological Park and Wildlife Rescue Center Kaski Nepal; ^6^ Florida Atlantic University Boca Raton Florida USA; ^7^ Central Department of Zoology Tribhuvan University Kathmandu Nepal; ^8^ Auckland College of Tertiary Studies CC Training Academy Auckland New Zealand

**Keywords:** elephant, endangered species, leopard, rhino, tiger, wildlife conservation, wildlife management, wildlife mortality

## Abstract

Wildlife conservation in human‐dominated landscapes faces increased challenges due to rising conflicts between humans and wildlife. We investigated the human and wildlife loss rates due to human–wildlife conflict between 2000 and 2020 in Nepal. We concentrated on Asian elephant (*Elephas maximus*), greater one‐horned rhino (*Rhinoceros unicornis*), tiger (*Panthera tigirs*), and leopard (*Panthera pardus*) mortality, as well as human mortality caused by these species. Over the 21‐year period, we recorded 1139 cases of wildlife mortality and 887 cases of human mortality. Leopard mortality was the highest, followed by that of greater one‐horned rhinos, tigers, and Asian elephants. Overall, the rate of wildlife mortality has been increasing over the years. Asian elephants were found to be more responsible for crop damage than greater one‐horned rhinos, while leopards were found to be more responsible for livestock depredation than tigers. The generalized linear model indicated that the mortality of wildlife in the districts is best predicted by the additive effect of human mortality, the proportion of agricultural land, and the literacy rate of the districts. Retaliatory wildlife mortality was the most challenging issue for wildlife conservation, especially for the large mammals. Findings from this study are important for mitigation of human–wildlife conflicts, controlling retaliatory killing, and conserving these threatened large mammals.

## Graphical abstract

We investigated the human and wildlife loss rate between 2000 and 2020 in Nepal. We focused on Asian elephant (*Elephas maximus)*, greater one‐horned rhino*(Rhinoceros unicornis)*,tiger (*Panthera tigris*), and leopard, *(Panthera pardus)* loss and human loss by those species. Overall, the rate of loss of wildlife and human was increasing over the years. Loss of wildlife was found to be the best predicted by additive effect of human loss, proportion of agricultural land, and literacy rate of the district.

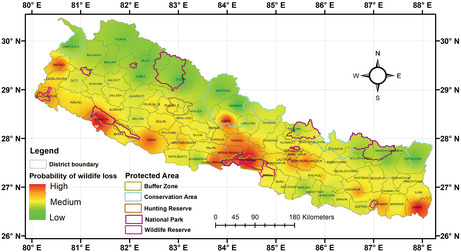

## 1 INTRODUCTION

Loss of wildlife due to retaliatory killing by people is one of the most critical issues for large mammal conservation (Adhikari, Baral, Bhandari, Szydlowski, et al., 2022; Baral, Aryal, et al., 2022; Bhandari & Chalise, 2016; Collins & Kays, 2011; Eklund et al., 2020; Nielsen et al., 2004). In areas where humans and wildlife share common resources (Bhandari, Adhikari, Baral, Panthi, et al., 2022; Nyhus, 2016; Rutina et al., 2016; Xu et al., 2019), the competition for limited resources results in a scarcity of food for wildlife in their natural habitats (Baral, Adhikari, & Bhandari, 2022). This may cause wildlife to expand their range to nearby villages and cause crop‐raiding, property damage, livestock depredation, and human attacks (Adhikari, Bhandari, Baral, Lamichhane, & Subedi, 2022; Baral, Sharma, Kunwar, et al., 2021). Such human–wildlife conflicts (HWC) often lead to people's negative attitudes toward wildlife conservation (Adhikari, Baral, Bhandari, Kunwar, & Subedi, 2022; Baral, Aryal, et al., 2022; Bhandari & Chalise, 2016). People may retaliate these losses by killing the wildlife involved in such conflicts as a form of retaliation killing (Adhikari, Bhandari, Baral, Lamichhane, & Subedi, 2022; Baral, Aryal, et al., 2022; Baral, Sharma, Rimal, et al., 2021; Gubbi et al., 2014; Nyirenda et al., 2015). The loss of wildlife due to poaching and trading (Everatt et al., 2019; Rosen & Smith, 2010) as well as HWC‐related wildlife mortalities significantly contribute to the fall in their populations.

Human–wildlife conflict is also accelerated due to an increment of human infrastructure development (such as road and building construction) in the wildlife habitats. Human settlements near wildlife habitats have resulted in a decrease in wildlife habitat size. Such encroachment of natural habitat and lack of conservation knowledge in the community (Nielsen et al., 2004; Pandey et al., 2016; Sijapati et al., 2021) has led to the population decline of several threatened species (Baral & Heinen, 2007; Stclair et al., 2019). Conflict between humans and wildlife in Nepal is higher in lowland regions where most of the large herbivores and carnivores are present (Acharya et al., 2016; Baral, Aryal, et al., 2022; Bhandari, Aryal, & Shrestha, 2019). However, the conflict between humans and large carnivores has been escalating in the mid‐hill areas in recent days (Adhikari, Bhandari, Baral, Lamichhane, & Subedi, 2022; Baral, Aryal, et al., 2022; Bhandari, Aryal, & Shrestha, 2019). People's high degree of dependency on forest products and livestock has led to frequent encounters with wildlife in these areas (Baral, Sharma, Rimal, et al., 2021; Kutal et al., 2021).

In Nepal, four large mammals, Asian elephants (*Elephas maximus*) (hereafter elephants), greater one‐horned rhinos (*Rhinoceros unicornis*) (hereafter rhinos), tigers (*Panthera tigris*) and leopards (*Panthera pardus*), are more involved in conflicts with people, including property damage and human casualties (Acharya et al., 2016; Baral, Aryal, et al., 2022; Bhandari, Mawhinney, et al., 2019; DNPWC, 2017; Pant et al., 2016; Thapa, 2014a). Among the four species, elephants, rhinos, and tigers are distributed in lowland areas while leopards are distributed throughout the country (Baral, Aryal, et al., 2022; Bhandari, Mawhinney, et al., 2019). Elephants and rhinos are usually involved in crop‐raiding, whereas carnivores such as tigers and leopards are involved in livestock predation and attacks on humans (Bhandari et al., 2021; Bhattarai & Fischer, 2014; Gurung et al., 2008; Sijapati et al., 2021). The population of large mammals such as tigers, rhinos, and elephants are increasing in Nepal. The tiger population has increased from 121 in 2009 to 355 in 2022 (DNPWC and DFSC, 2022). Similarly, the number of rhinos also increased from 605 in 2015 to 752 in 2021 (DNPWC, 2021). The population of elephants and leopards has also been believed to be increasing in recent years, but the population census has not been assessed till date (DNPWC, 2017, 2021; DNPWC and DFSC, 2022; Ram et al., 2021). It is assumed that establishment of community forest programs and conservation awareness etc. could contribute to increasing population (Baral, Sharma, Kunwar, et al., 2021). Escalation of conflicts is inevitable in the scenario of an increasing number of large mammals (Mukeka et al., 2019). However, HWC has also increased due to encroachment of forest land, habitat fragmentation, and inefficient wildlife management practices in the regions outside protected areas (PAs) of Nepal (Baral, Sharma, Rimal, et al., 2021).

The government of Nepal has implemented various species‐specific management strategies and a wildlife damage compensation scheme to minimize HWC. The HWC mitigation program was formerly started in PAs in 1996 and outside PAs in 2012 (Acharya et al., 2016; Bhattarai & Fischer, 2014). Despite the conservation efforts, new incidents of wildlife killing have been common over the years (Bhandari et al., 2019; Dhungana et al., 2018; Pant et al., 2016). Wildlife mortality is a prominent issue that has to be resolved for large mammal conservation in human‐dominated landscapes (Baral & Heinen, 2007; McGuinness & Taylor, 2014; Santiapillai et al., 1982, 2010; Zhang & Wang, 2003). Moreover, it is necessary to understand the patterns of human and wildlife mortality over a longer period in order to understand the trend and drivers of HWC, as such research provides foundational data for formulation and implementation of sustainable conservation strategies. Because large mammals are more likely to be involved in HWC events (Adhikari, Baral, Bhandari, Kunwar, & Subedi, 2022; Baral, Sharma, Rimal, et al., 2021), our study had two major goals: (1) to identify trends in human–large mammal conflict over two decades, and (2) to examine major attributes associated with large mammal mortality over two decades. We hypothesized the increasing trend of human and large mammal casualties and the mortality of human to be a significant variable for predicting the mortality of large mammals.

## 2 MATERIALS AND METHODS

### 2.1 Data collection

The study was conducted in Nepal (26.36–30.45° N, 80.06–88.2° E). A total of 192 species of mammals are found in Nepal (Thapa, 2014b), encompassing about 3.5% of the world's mammalian fauna. Our data collection was focused on records of the mortality of four large mammals: elephants, rhinos, tigers, and leopards, due to the prominent number of human–wildlife conflicts associated with these four species. We also recorded the data regarding the mortality of human life and property damage (such as livestock loss and crop depredation) caused by these four species. Data were gathered from government reports [Department of National Parks and Wildlife Conservation (DNPWC), Department of Forest and Soil Conservation (DoFSC), Division Forest Offices (DFO), and Protected Areas (PAs)], technical reports, and newspapers published between 2000 and 2020 (See Data Availability Statement). During the collection of data, we recorded the nature of events (human mortality, wildlife mortality, livestock loss, and extensive crop loss), species involved in conflict, year, and location. Duplications and redundancies were crosschecked. Moreover, reliable records, such as the verified reports of government agencies, i.e., DNPWC, DoFSC, DFOs, and PAs were used for the purpose of validation, and the incomplete and unreliable records were omitted from the analysis.

### 2.2 Data analysis

We applied generalized linear models (GLM) with a Gaussian distribution to understand the influence of independent variables on wildlife mortality in 77 districts of Nepal (Dobson & Barnett, 2018; Hardin et al., 2007). The number of wildlife mortality (Wloss) in a particular district was set as a dependent variable, while seven independent variables: (1) number of human losses within districts (Hloss), (2) proportion of agricultural land within districts (Pag), (3) literacy rate of people in the district (Lrate), (4) population density within district (Pden), (5) proportion of forest land within district (Pfr), (6) proportion of protected area within district (Ppa), and (7) density of livestock within district (Lden) were included in the analysis. All of the variables were included in further modeling because they had Pearson's correlations of |*r*| < .7.

We used Akaike's Information Criterion; corrected for small sample sizes (AICc), to perform model selection. We selected the most parsimonious models based on a delta AIC < 2 and extracted the model averaged results to calculate parameter estimates using the MuMIn package (Barton & Barton, 2015) in R program. A total of 127 models with different combinations of seven independent variables were analyzed. Out of these models, seven models (Appendix 1) performed the best with a delta AIC value <2. We obtained model averaged coefficients using the seven best‐fit models, and the significant predictor variables (*p* < .05) from the best‐fit model were used to generate the probability of wildlife mortality over two decades. The Inverse Distance Weighing (IDW) function in ArcGIS was used to generate the heatmap (Al‐Bakri et al., 2011; Kunwar et al., 2022). The IDW interpolator assumes that the local influence that each point possesses diminishes with distance, resulting in the decreasing influence of a variable with increasing distance from its sampled location (Shepard, 1968). It also attributes greater weight to the points that are closer to the processing cell than those that are further away. The interpolated surface, estimated through the moving average technique, generates values less than the local maximum and greater than the local minimum (Shepard, 1968; Wong, 2016). The in‐point features were represented by 77 districts, and the z field represented numerical values of wildlife loss. The exponent of distance, which controls the significance of surrounding points on the interpolated value, was set as 2 (the default value). We used variable search radius, whereas the maximum distance to limit the nearest search sample was also set to the default value (the length of the extent's diagonal). The procedure of interpolation was carried out using the spatial analysis technique within ArcGIS (ESRI, 2011). Finally, the heatmap was reclassified by assigning the values as low probability (0–0.40), medium probability (0.40–0.60), and high probability (0.60–1) of wildlife mortality.

## 3 RESULTS

**FIGURE 1:**
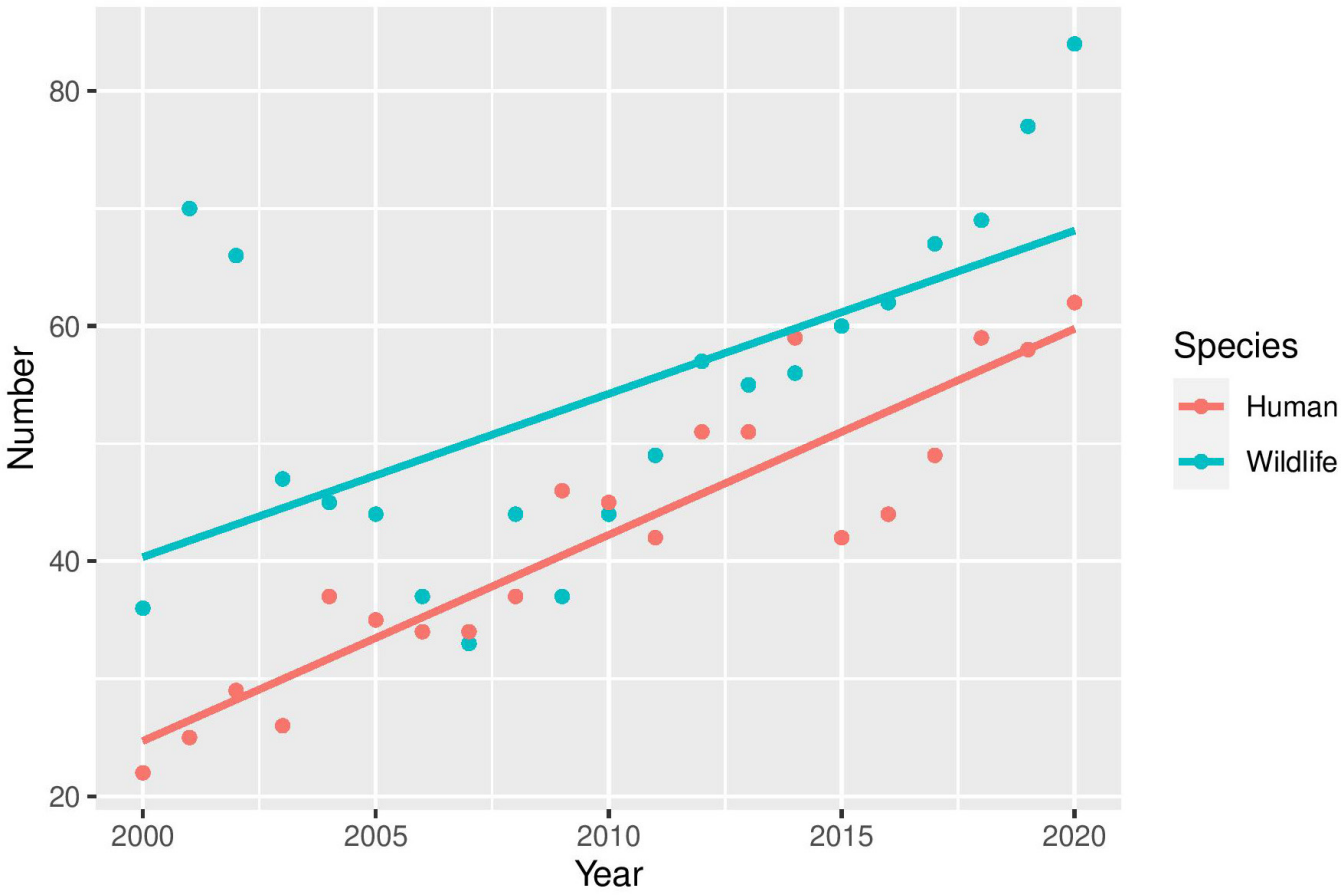
The trend of large mammal loss and human loss from large mammals between 2000 and 2020 in Nepal.

**FIGURE 2:**
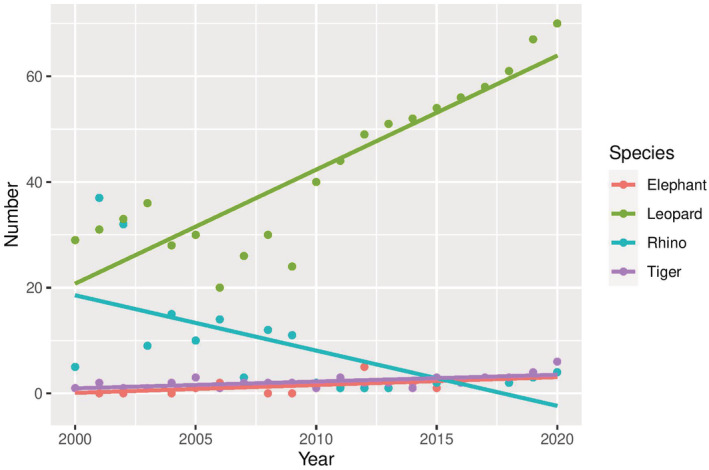
Patterns of large mammal loss due to human–wildlife conflict between 2000 and 2020 in Nepal.

**FIGURE 3:**
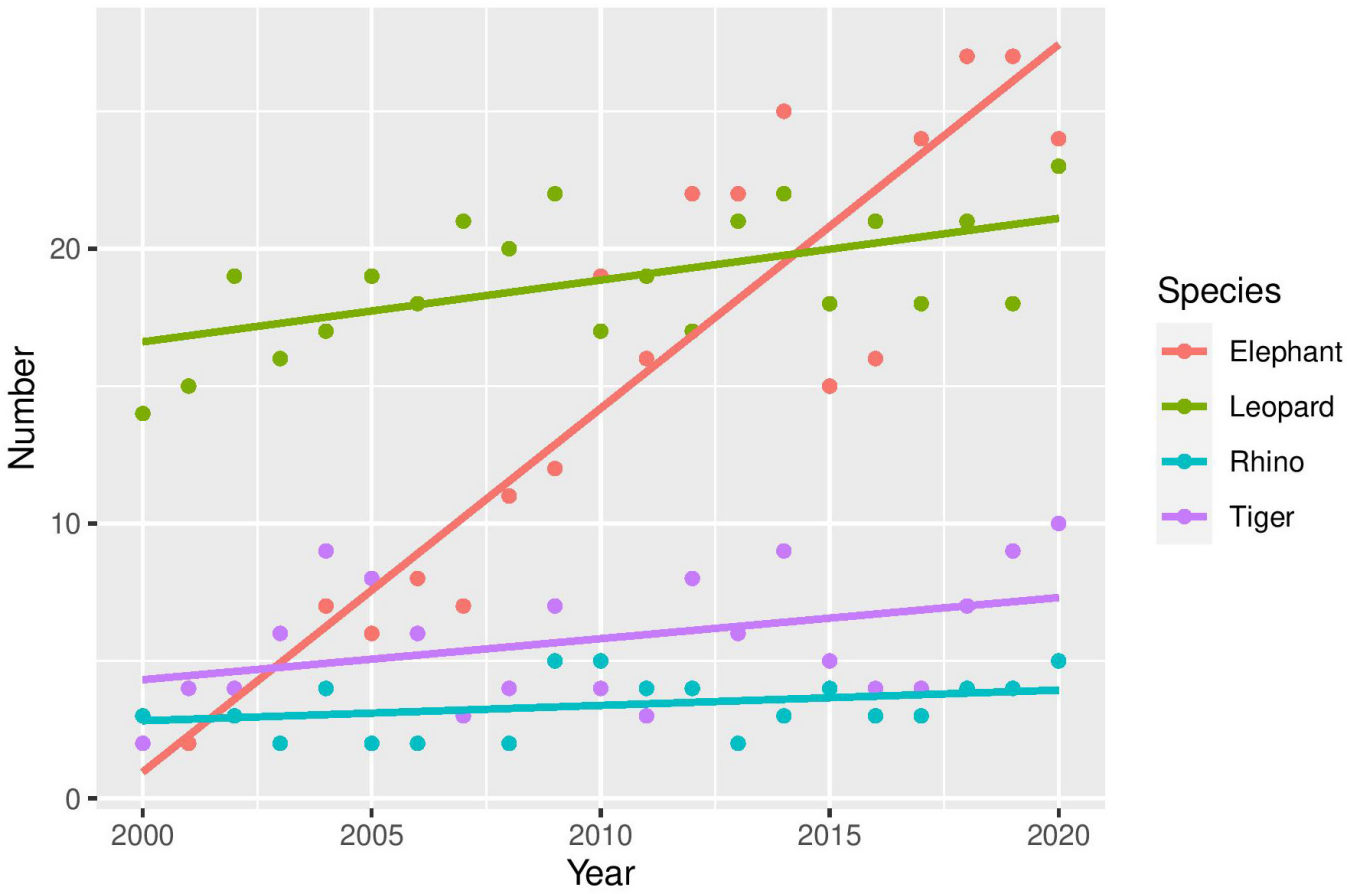
Patterns of human loss from the attacks of four large mammals between 2000 and 2020 in Nepal.

**FIGURE 4:**
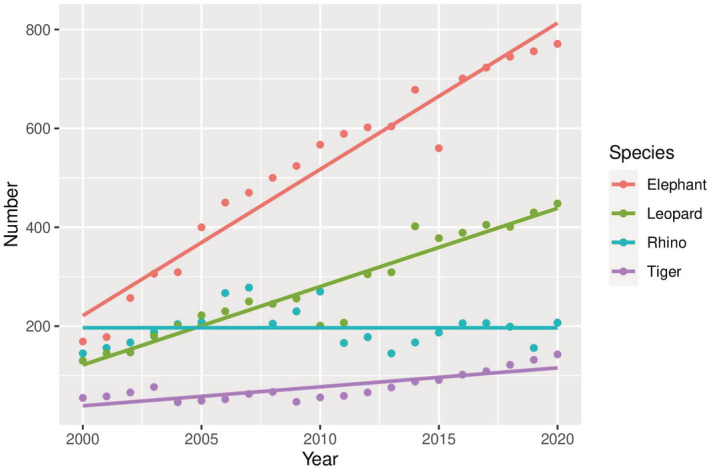
Total number of events of property damage (crop depredation by elephants and rhinoceros, and livestock predation by tigers and leopards) between 2000 and 2020 in Nepal.

We recorded 1139 (x̄ 54.2/year, SD ± 14.3) HWC‐related large mammal losses and 887 (x̄ 42.2/year, SD ± 11.8) human losses between 2000 and 2020 in Nepal (Figure 1). Out of four species, leopard loss was the highest (x̄ 42.3/year, SD ± 15.1), followed by rhinos (x̄ 8.0/year, SD ± 6.9), tigers (x̄ 2.2/year, SD ± 1.2), and elephants (x̄1.5/year, SD ± 1.3) (Figure 2). During this period, leopards killed an average of 18.8 people (SD ±2.4) yearly, followed by elephants at 14.1 (SD ± 8.8), tigers at 5.8 (SD ± 2.3), and rhinos at 3.3 (SD ± 1.1) (Figure 3). Overall, the rate of wildlife mortality was higher compared with human mortality. Both the HWC related wildlife and human mortality have shown an increasing trend with time (wildlife mortality *R* ^2^ = 0.35, human mortality *R* ^2^ = 0.84) (Figure 1). The results showed a high correlation (*r* = .44) between wildlife and human mortality over the period of 21 years. We recorded 517 (SD ± 188.7) events of crop damage by the elephant, followed by the rhinos with 280 events (SD ± 39) yearly. Similarly, leopards contributed the highest livestock depredation (280 livestock/year, SD ± 103.3) followed by tigers (77 livestock/year, SD ± 28.9) (Figure 4).

**FIGURE 5:**
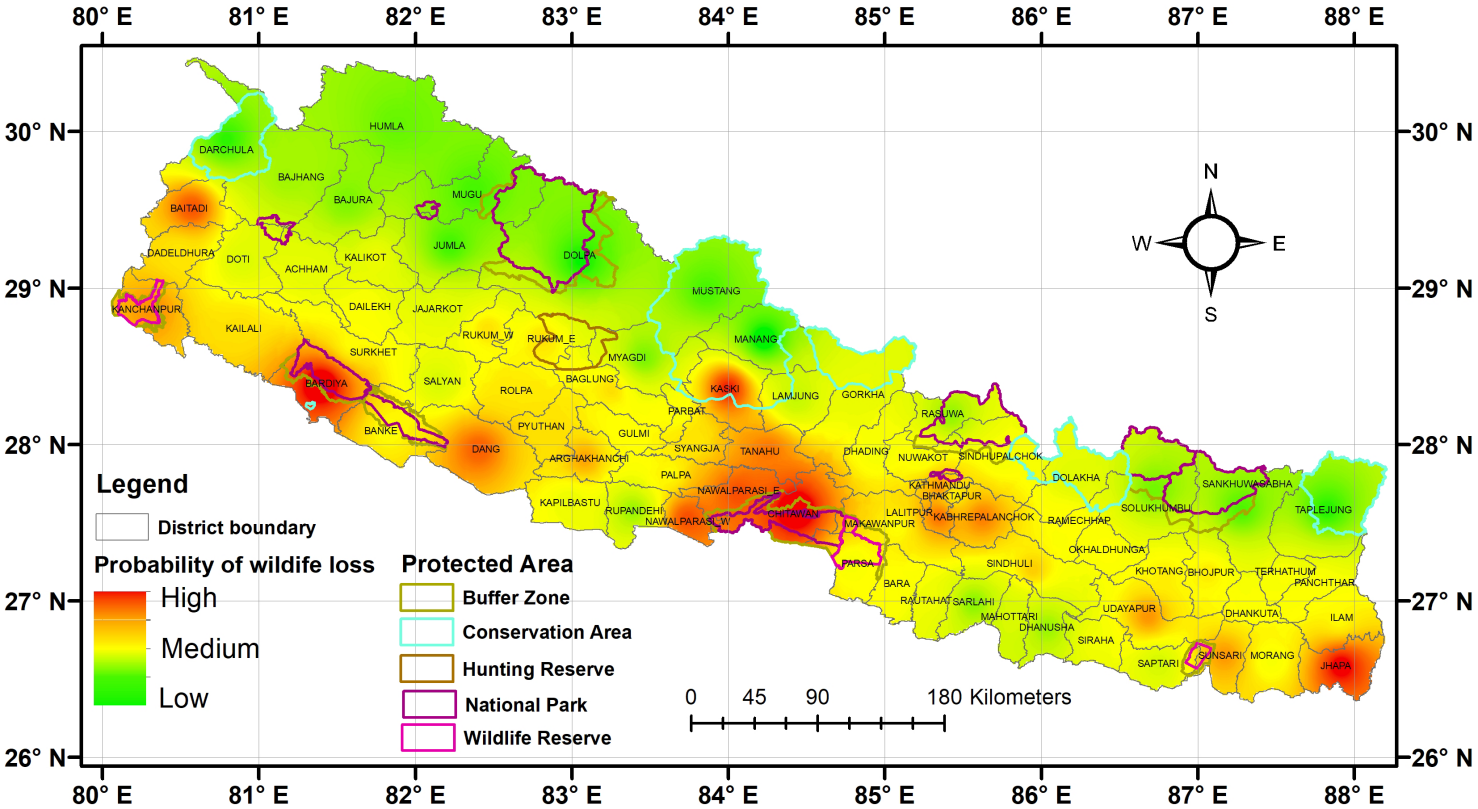
A map depicting the likelihood of wildlife loss based on model averaged coefficients of the best fit models. Green represents a low probability of wildlife loss (0–0.40), yellow a medium probability (0.40–0.60), and red a high probability (0.60–1).

The model with the highest AIC weight (0.119) to predict wildlife mortality was the model with the additive effect of three variables (number of human losses + proportion of agricultural land within district + literacy rate of district). The average of seven best fit‐models (with delta AICc < 2) (Appendix 1) found that the number of human losses and proportion of agricultural lands have significant positive associations with the number of wildlife mortality, whereas literacy rate had a significant negative association (Table 1). We found a high probability of wildlife loss in lowland districts such as Jhapa, Chitwan, Nawalparasi, Dang, Bardia, and Kanchanpur. Similarly, the mid‐hill districts with a high probability of wildlife mortality were Kabhre, Kathmandu, Lalitpur, Bhaktapur, Tanahun, Kaski, Argakhachi, and Baitadi (Figure 5). Most of the mid‐hill districts were under medium risk of wildlife mortality, whereas the high mountain and high Himalayan regions had a lower probability of wildlife mortality. The heat map depicted central Nepal as the most prominent zone for HWC‐related wildlife mortality (Figure 5).

**TABLE 1:** Model averaged coefficient for the seven best‐fit models (delta AIC < 2), where: number of wildlife losses: (Wloss), number of human losses within districts: (Hloss), proportion of agricultural land within district: (Pag), literacy rate of people in district: (Lrate), population density within district: (Pden), proportion of forest land within district: (Pfr), proportion of protected area within district: (Ppa), livestock density within district: (Lden).

Variables	Estimate	Std. Error	Adjusted SE	*z* value	Pr(>|*z*|)	Significance value
(Intercept)	0.001373	0.027441	0.027908	0.049	0.96075	
Hloss	0.963708	0.033709	0.034248	28.139	<2e‐16	***
Pag	0.079198	0.038371	0.038938	2.034	0.04196	*
Lrate	−0.101064	0.032002	0.032499	3.11	0.00187	**
Pden	0.053734	0.033577	0.034092	1.576	0.11499	
Pfr	0.033057	0.031325	0.03185	1.038	0.29932	
Ppa	−0.028436	0.029069	0.029566	0.962	0.33616	
Lden	−0.031387	0.052179	0.053071	0.591	0.55425	

*Note*: *p = .05, **p < .001, ***p < .0001.

## 4 DISCUSSION

Our results shed light on an important component of HWC and reveal that the HWC‐related wildlife mortality in Nepal is escalating, and is largely associated with human casualties, the proportion of agricultural area and the literacy rate of people within districts. The correlation between wildlife and human mortality indicates that retaliatory actions (from humans) play an important part in wildlife mortality.

The incidence of casualties is particularly common in human‐dominated areas due to shared use of resources (Acharya et al., 2016; Bhandari, Mawhinney, et al., 2019). Among different types of HWC, crop raiding, livestock loss, and human loss are the most prominent causes (Acharya et al., 2016; Baral, Sharma, Rimal, et al., 2021; Bhandari & Chalise, 2016). Similarly, wildlife killing as a retaliatory action against crop loss and livestock depredation is one of the major reasons for wildlife death (Baral, Aryal, et al., 2022; Bhandari & Chalise, 2016; Karanth et al., 2019; Mateo‐Tomás et al., 2012). The correlation between the proportion of agricultural land and retaliatory killing of wildlife confirms such a trend. The greater the agriculture land near the forest, the greater the chance of crop and livestock loss by wildlife. Consequently, these species are highly victimized by local people, mostly farmers. Moreover, the lower literacy level of the people is also responsible for the higher rate of wildlife killing. Illiterate people may have lower awareness of the ecological value of wildlife and are more likely to retaliate when their livelihood is damaged by wildlife (Bhandari, Mawhinney, et al., 2019). Similarly, illiterate people usually sustain their lifestyle with a higher dependency on agricultural practices and livestock rearing activities in rural areas, which increases the possibility of their encounter with wildlife. This encounter likely results in casualties of wildlife due to retaliatory action of those people in response to livestock/property loss.

Our results showed that a majority of human losses were primarily attributed to the attacks by leopards in the mid‐hill physiographic region of Nepal. A high number of human casualties due to leopard attacks was reported in the central mid‐hills of Nepal, resulting in an average of 12 leopards being killed annually in retaliation between 2015 and 2019 in the same region (Baral et al., 2021). These higher conflicts between humans and leopards could be primarily associated with lower prey availability in the forest (Sharma et al., 2021). Leopards' primary prey species are ungulates (i.e. barking deer and wild boar), and their populations in the mid‐hills have declined significantly in recent years (Baral et al., 2021, 2022). Moreover, people in mid‐hill areas are mostly farmers with agro‐pastoralism, with forest‐dependent livelihood, and cattle herding practices, which can lead to increased leopard mortality due to retaliatory actions. At the same time, with the increasing human presence in and around the leopard's habitat for hunting, poaching, timber, and firewood collections, they also result in a higher rate of deliberate and fateful encounters between humans and leopards. Leopards generally inhabit mid‐hill regions (between 500 and 3000 m), and their population will likely decrease in the near future due to habitat loss, loss of prey, and habitat fragmentation (Baral, Sharma, Rimal, et al., 2021; Bhandari et al., 2021; Bhandari, Mawhinney, et al., 2019; Sharma et al., 2021). Such a situation is concerning not only for leopards but also for overall wildlife conservation (Acharya et al., 2016; Baral et al., 2021; Bhandari, Mawhinney, et al., 2019; Pant et al., 2016).

Livestock depredation, human casualties, and poaching for pelts (Bhattarai et al., 2019; Bhattarai & Fischer, 2014; Eklund et al., 2020) are regarded as some of the major causes for tiger mortality. Human deaths from tiger attacks were significantly higher near PAs than in other areas (Bhattarai & Fischer, 2014; Dhungana et al., 2018; Gurung et al., 2008). The trend of human deaths due to tiger attacks in places such as Chitwan National Park (CNP) has increased significantly, from an average of one person per year prior to 1998 to seven people per year after 2000 (Gurung et al., 2008). Over 70% of the tiger population refuges in the CNP and Bardiya National Park (BNP) (Bhandari, Aryal, & Shrestha, 2019). However, in recent times, tigers have been recorded even in the mid‐hill regions (Illam and Dadeldhura districts). This expansion of the range could be attributed to two major factors. First, the recent increase in the population of tigers could have potentially forced them to search for new habitats. Second, the restoration of north–south corridors and increased linkage between habitats in lowlands and uplands may have created a favorable scenario for tiger range expansion in the mid‐hills region. With the increased population of tigers in recent days and the expansion of tiger range, more conflict incidences can be presumed to follow in coming days if proper habitat management techniques (prey restoration, habitat management, etc.) are not efficiently implemented.

According to our research, anthropogenic factors are also contributing to the mortality of elephants and rhinos. The human‐caused mortality of elephants is also gradually increasing in Nepal. The average mortality rate for the elephant was 1.5 per year during 2000–2020. However, anthropogenic mortality of rhinos was found to be decreasing in recent years, with an average of eight rhinos killed per year during the study period. These species are also responsible for the killing of people. Elephant attacks killed 14.1 people on average per year, whereas rhino attacks killed 3.3 people. The incidence of human casualties attributed to elephant attacks has been surging rapidly in recent years (Ram et al., 2021). These large herbivores prefer feeding on diverse types of plant species as well as frequently visit human settlements and agricultural lands near protected areas to feed on crops. These incidences are quite common at CNP, BNP, Sukhaphanta National Park (SNP), and Koshi Tapu Wildlife Reserve (KWR) (Koirala et al., 2016; Short, 1981). This pattern of resource use near a human‐dominated landscape can lead to HWCs and may result in both human and wildlife casualties (Bhandari, Adhikari, Baral, & Subedi, 2022; Martin et al., 2009). In addition, these mega herbivores using farms, roads, and human trails for their movement may also damage infrastructure and increase HWC (Bhandari, Adhikari, Baral, & Subedi, 2022; Shaffer et al., 2019). Similarly, loss of the natural habitat of these mega herbivores and fragmentation of biological corridor forests in southern Nepal, especially for elephants, has aided in the escalation of human–elephant conflicts (Yadav, 2007). Nepal's lowland forest or Chure hill forest contributes as a major corridor for elephant migration, where elephants migrate between Nepal and India (Dhakal & Thapa, 2019; Pant et al., 2016). In most of these areas, elephant migratory routes have been destroyed and converted into villages and agricultural land, leading to increased human and elephant conflict. Human killings by rhino attacks were relatively lower than elephants. This may be because rhino populations are concentrated in a few pocket areas within Nepal, such as CNP and BNP. Most of the victims in CNP between 2003 and 2013, including fatalities and losses of property, were mostly associated with the crop loss by rhino (Ruda & Kolejka, 2020). Large amounts of crop damage may be one of the major causes of the killing of rhino (Bhandari, Adhikari, Baral, & Subedi, 2022). Similarly, poaching could contribute to rhino mortality. However, there has been a decrease in poaching in recent years, but it has not stopped completely.

The heatmap created using the coefficients from the best‐fit models revealed that the southern lowland districts (i.e. Kanchanpur, Bardiya, Dang, Nawalparasi, Chitwan, Sunsari, and Jhapa) have the highest likelihood of loss for these large mammals. The fact that these habitats are home to all four large mammals may be one of the major factors contributing to the higher likelihood of wildlife mortality in this area. The higher percentage of agricultural lands in these districts and the greater number of human casualties could be considered additional factors. While several mid‐hill districts (i.e. Baitadi, Argakhachi, Kaski, Tanahun, Kathmandu, and Lalitpur) had a higher likelihood of mortality, the majority of the mid‐hills showed a medium probability of wildlife mortality. The loss of wildlife in the mid‐hills is mostly represented by the events of leopard loss. Our study supported the findings of previous studies as high human–leopard conflict is frequently reported from the mid‐hill areas (Acharya et al., 2016; Adhikari, Baral, Bhandari, Kunwar, & Subedi, 2022; Baral et al., 2021; Pant et al., 2016). On the contrary, the high Himalayan regions had a very low probability of wildlife loss, particularly because the studied species are largely absent in these areas.

On the basis of a secondary database of HWC on Nepal over two decades, our study illustrated the overall scenario of the human–large mammal conflict with a focus on the attributes of anthropogenic mortality of four threatened species within Nepal. However, a large number of HWC events may go unreported to authorities and thus be absent from the database. Hence, the actual number and incidences of conflicts could be potentially higher than the reported cases. For example, tigers, leopards, elephants, and rhinos are among the protected wild species of Nepal. As killing those species is illegal, people may hide such information. Similarly, in some remote areas in Nepal, where there is limited access to communication, HWC incidents might not be reported to the authorities. Nevertheless, our study included vital HWC information for over two decades, gathered from reliable sources, therefore, these results represent the overall scenario of human–large mammal conflict in Nepal.

## 5 CONCLUSIONS

This study concluded that anthropogenic mortality of large mammals has been increasing in recent years, posing a challenge to the conservation of threatened species such as the tiger, elephant, rhino, and leopard. Managing human–large mammal conflict and mitigating retaliatory killing of those species is particularly challenging due to retaliatory emotions associated with human casualties, livestock losses, and property damage. Mortality of large mammals increased with the number of human casualties and the proportion of agricultural land within the district, whereas it decreased in the districts with a higher literacy rate. In order to reduce the mortality of large mammals, potential retaliatory actions by local people should be strictly controlled. Similarly, habitat fragmentation should be controlled, and local people should be motivated to inform the conservation authorities in the event of conflict. Extension of conservation education and conflict mitigation programs among high wildlife mortality districts is imperative for sustained wildlife conservation. At the same time, the database on human casualties and wildlife loss should be upgraded at the local and national level and integrated into national and provincial level planning, particularly outside PAs.

## AUTHOR CONTRIBUTIONS

**Kedar Baral:** Conceptualization (lead); data curation (equal); formal analysis (lead); methodology (lead); visualization (lead); writing – original draft (lead); writing – review and editing (lead). **Shivish Bhandari:** Data curation (equal); formal analysis (equal); writing – review and editing (equal). **Binaya Adhikari:** Data curation (equal); formal analysis (equal); writing – review and editing (equal). **Ripu M. Kunwar:** Supervision (equal); writing – review and editing (equal). **Hari P. Sharma:** Supervision (equal); writing – review and editing (equal). **Achyut Aryal:** Supervision (equal); writing – review and editing (equal). **Weihong Ji:** Supervision (lead); writing – review and editing (equal).

## CONFLICT OF INTEREST

None declared.

## Data Availability

The dataset that is associated with this study is available at dryad (https://doi.org/10.5061/dryad.1jwstqjz8).

## APPENDIX 1

### Seven best fit models (with delta AIC < 2) out of 127 models ran in the analysis, where, number of wildlife losses: (Wloss), number of human losses within districts: (Hloss), proportion of agricultural land within district: (Pag), literacy rate of people in the district: (Lrate), population density within district: (Pden), proportion of forest land within district: (Pfr), proportion of protected area within district: (Ppa), density of livestock within district: (Lden).

**Table:**

Models	df	logLik	AICc	delta	weight
Wloss~Hloss + Pag + Lrate	5	−127.33	265.5	0	0.24
Wloss~Hloss + Pag + Pden + Lrate	6	−126.35	265.91	0.41	0.19
Wloss~Hloss + Pag + Pfr + Pden + Lrate	7	−125.55	266.73	1.23	0.13
Wloss~Hloss + Pden + Lrate	5	−127.99	266.83	1.33	0.12
Wloss~Hloss + Pag + Pfr + Pden + Lden	6	−126.82	266.84	1.34	0.12
Wloss~Hloss + Pag + Pfr + Pfr + Lrate	6	−126.9	267	1.5	0.11
Wloss~Hloss + Pag + Pfr + Lden + Lrate	6	−127.13	267.47	1.97	0.09
